# The reversal of pulmonary vascular remodeling through inhibition of p38 MAPK-alpha: a potential novel anti-inflammatory strategy in pulmonary hypertension

**DOI:** 10.1152/ajplung.00038.2015

**Published:** 2015-05-29

**Authors:** Alistair C. Church, Damien H. Martin, Roger Wadsworth, Gareth Bryson, Andrew J. Fisher, David J. Welsh, Andrew J. Peacock

**Affiliations:** ^1^Scottish Pulmonary Vascular Unit, University of Glasgow, Glasgow, United Kingdom;; ^2^Department of Cardiovascular Biology, University of Strathclyde, Glasgow, United Kingdom;; ^3^Department of Pathology, Southern General Hospital, Glasgow, United Kingdom; and; ^4^Institute of Cellular Medicine, Newcastle University, Newcastle upon Tyne, United Kingdom

**Keywords:** pulmonary hypertension, p38 MAPK

## Abstract

The p38 mitogen-activated protein kinase (MAPK) system is increasingly recognized as an important inflammatory pathway in systemic vascular disease but its role in pulmonary vascular disease is unclear. Previous in vitro studies suggest p38 MAPKα is critical in the proliferation of pulmonary artery fibroblasts, an important step in the pathogenesis of pulmonary vascular remodeling (PVremod). In this study the role of the p38 MAPK pathway was investigated in both in vitro and in vivo models of pulmonary hypertension and human disease. Pharmacological inhibition of p38 MAPKα in both chronic hypoxic and monocrotaline rodent models of pulmonary hypertension prevented and reversed the pulmonary hypertensive phenotype. Furthermore, with the use of a novel and clinically available p38 MAPKα antagonist, reversal of pulmonary hypertension was obtained in both experimental models. Increased expression of phosphorylated p38 MAPK and p38 MAPKα was observed in the pulmonary vasculature from patients with idiopathic pulmonary arterial hypertension, suggesting a role for activation of this pathway in the PVremod A reduction of IL-6 levels in serum and lung tissue was found in the drug-treated animals, suggesting a potential mechanism for this reversal in PVremod. This study suggests that the p38 MAPK and the α-isoform plays a pathogenic role in both human disease and rodent models of pulmonary hypertension potentially mediated through IL-6. Selective inhibition of this pathway may provide a novel therapeutic approach that targets both remodeling and inflammatory pathways in pulmonary vascular disease.

pulmonary hypertension continues to be a cause of significant morbidity and mortality and there remains a need for novel approaches in tackling the underlying pulmonary vascular remodeling (PVremod). This remodeling is characterized by increased proliferation of vascular cells, increased extracellular matrix and collagen deposition, recruitment of bone marrow-derived cells to the vascular wall, and increased inflammatory responses (4). Although inflammation in driving this process is now recognized, with, for example, interleukin-6 (IL-6) being shown to have an important pathogenic role, the exact link between inflammation and pulmonary vascular remodeling remains unclear (2, 15).

Although both smooth muscle and endothelial cells are vital to the pathology of pulmonary hypertension, the fibroblast plays a critical role in PVremod by undergoing proliferation in response to many stimuli, for example, hypoxia, and driving smooth muscle proliferation (13). In addition, it has been established by our own group and others that the adventitial fibroblast can contribute significantly to inflammatory processes and is a source of cytokines and chemokines, which can recruit key inflammatory cells such as monocytes/macrophages to the sites of PVremod (7, 17, 20). Previous work from our group has demonstrated in vitro that activation of the p38 mitogen-activated protein kinase (MAPK) pathway is essential to fibroblast proliferation in the hypoxic model and that its inhibition can block the hypoxic proliferative response (11, 21). In addition, p38 MAPK is well established in other inflammatory pathological states as being a key intermediary pathway and plays an important role in mediating cellular responses to proinflammatory cytokines. Phosphorylation and activation of the alpha (α)-isoform of p38 MAPK have been implicated in both driving the hypoxic proliferation of the adventitial fibroblast (9) and release of mitogens from the fibroblast that stimulate adjacent smooth muscle cell proliferation and recruit proinflammatory cells (13, 21). Fibroblast proliferation is an important mechanism underpinning PVremod and at least in hypoxia is p38 MAPK dependent (14). This contrasts with the systemic vascular fibroblast, which does not proliferate nor does it upregulate the p38 MAPK pathway in hypoxia, offering the possibility of selectively targeting the abnormal pulmonary vascular tree in pulmonary hypertension with minimal effect on systemic vessels (12). In addition p38 MAPK has been implicated in the development of pulmonary hypertension in other animal models, although no clear mechanism has been suggested. Although both inflammation and p38 MAPK have been implicated in PVremod a possible link between the two in pulmonary hypertension has not previously been suggested. This study sought to investigate the hypothesis that p38 MAPK, and the α-isoform in particular, mediates PVremod and pulmonary hypertension through an inflammatory process by employing both prevention and reversal inhibitor strategies in vivo and exploring p38 MAPK expression in human disease.

## METHODS

### Primary Cell Culture

Fibroblasts from adult male Sprague-Dawley rats were obtained from freshly excised vessels using a modified explant technique, as previously described (10). Briefly, vessels were dissected free and opened into a flat sheet and muscular tissue and endothelial cell layers were removed by gentle abrasion. The remaining tissue (adventitia) was then dissected into 1-mm^3^ portions, ∼25 of which were evenly distributed over the base of a 25-cm^2^ culture flask containing 2 ml of DMEM with 20% fetal calf serum (FCS), penicillin/streptomycin (400 IU/ml and 400 mg/ml), amphotericin B (5 mg/ml), and l-glutamine (27 mg/ml). Explants were incubated in a humidified atmosphere of 5% CO_2_ in air at 37.0°C. Although there are no specific fibroblast markers, we have previously shown using cell morphology and the absence of smooth muscle actin that this technique produces a pure culture of fibroblasts (21). To further characterize the cell population, vimentin staining was also used. Cells were subsequently maintained in DMEM containing 10% FCS, supplemented with antibiotics and l-glutamine as before, and used between passage 3 and 10. All chemicals were obtained from Invitrogen (Paisley) unless otherwise stated.

### Growth of Cells in a Hypoxic Environment

Maintenance of cells in a nitrogen-supplemented, humidified, temperature-controlled incubator (Galaxy R; Wolf Laboratories, York, UK) allowed control of internal chamber oxygen levels at 5%, while CO_2_ level was maintained at 5%. This achieved a tissue culture supernatant Po_2_ of 35 mmHg for the hypoxic conditions (10).

### Animal Models

Adult male Sprague-Dawley rats, weighing 200–250 g, were used in all experiments. All experiments were approved by the University of Strathclyde Ethics Committee and were performed under authorization of the Animals (Scientific procedures) Act 1986.

#### Chronic hypoxic model.

Animals were maintained in a hypobaric chamber at ambient pressure of 550 mmHg for up to 4 wk, equivalent to an oxygen concentration of 10%. The chamber was depressurized initially over 2 days. To allow daily injections the chamber was brought back to atmospheric pressure over a 1-h period and injections performed and then the chamber was depressurized again with gradual reductions over 1 h. The chamber was unsealed for a maximum of 30 min/day. Age and weight matched normoxic animals were housed in an adjacent perspex container but without depressurization. Animals received food and water ad libitum. Animals body weights were measured twice weekly.

#### Monocrotaline model.

Monocrotaline (MCT) was dissolved in 1 M HCl and then neutralized with a similar volume of 1 M sodium hydroxide. Animals were then injected with 60 mg/kg MCT subcutaneously in the scruff of the neck. Control animals received a similar volume of saline injected subcutaneously. The animals body weights were measured three times a week. The agents used were SB203580 and PH-797804. Both are p38 MAPK α-specific but the PH-797804 has been shown to be more specific and tolerated in humans.

The animal groups were divided up into: *1*) a prevention study for chronic hypoxia where the p38 MAPK antagonists were administered at the start of the animals being exposed to the hypobaric chamber; *2*) a reversal study for chronic hypoxia where the animals were allowed to develop pulmonary hypertension for 2 wk and were then treated with SB203580, a p38 MAPK antagonist; *3*) a reversal study for the MCT-treated animals with the p38 MAPK antagonist SB203580 as above; *4*) a reversal study with the p38 MAPK antagonist PH-797804 for the chronic hypoxic animals; and *5*) a reversal study with the p38 MAPK antagonist PH-797804 for the MCT animals.

### Measurement of Hemodynamics and Right Ventricular Hypertrophy

Rats were anesthetized using subcutaneous administration of a 50:50 mixture of midazolam (Hypnovel) and fentanyl/flunisone (Hypnorm) at a dose of 0.15 ml per 100 g body wt. The animals underwent tracheotomy and were established on a ventilator. Then a midline thoracotomy was performed and the heart was exposed. The right ventricular (RV) pressure was obtained by advancing a 21-G needle attached to a pressure transducer into the RV. The pressures were recorded using a Powerlab data acquisition system (AD Instruments) and analyzed using LabChart software. Placement of the catheter in the RV was established by direct visualization and afterwards by postmortem examination. The tracings were allowed to stabilize for 3 min.

#### Systemic blood pressure.

This was measured by noninvasive tail-cuff methods as previously outlined (26).

#### Assessment of right ventricular hypertrophy.

The RV was separated from the left ventricle (LV) and septum (S) using a dissecting microscope. The ventricles were blotted and then weighed. The degree of RV hypertrophy (RVH) was determined by the ratio of the weight of the RV to the LV + S.

#### Thymidine proliferation assay.

Rat pulmonary artery fibroblasts (RPAFs) were grown in normoxic conditions to 60% confluency in 24-well plates and then quiesced for 24 h in serum free medium. Cells were then cultured under conditions of normoxia or hypoxia, in the presence or absence of serum and experimental mediators, for a further 24 h. Increased proliferation of RPAFs to hypoxia is seen with concentrations of serum 0.1%–10%. For these experiments, 1% serum was selected as this maximizes differences between normoxic and hypoxic proliferation.

Fibroblasts were pulsed with [^3^H]thymidine (0.1 μCi/well) 4 h before the end of the 24 h experimental period with scintillation counting as previously described. To assess proliferation and viability 24 h, experiments were repeated, and fibroblasts were removed from the wells by 0.05% trypsin and then counted using a Fischer hemocytometer/0.4% trypan blue.

#### Immunoblotting.

Frozen lung tissue was homogenized on ice using a rotor blender (Fisher) on ice in Tissue protein extraction reagent (*T-Per* from Sigma). This was supplemented with phosphatase and protease inhibitors (Halt; Sigma). Homogenates were then centrifuged for 15 min at 4°C, and the supernatants were collected and frozen at −80°C until required. The protein concentration was established using a BCA technique (Thermo Scientific), and 30–40 μg of protein were then separated by electrophoresis on a Bis-Tris NuPage gel. Proteins were then transferred to PVDF Immobilon and transfer was confirmed with Ponceau red stain. The blot was blocked at room temperature for 1–2 h in 5% nonfat milk in Tris-buffered saline containing 0.05% Tween-20. Membranes were then incubated overnight at 4°C with primary antibody diluted accordingly in 5% milk/TBS-T. These were subsequently washed using TBS-T and then incubated with secondary antibody for 1–2 h at room temperature. The antibody labeling was visualized using enhanced chemiluminscence (ECL; Amersham) with exposure to autoradiographic film (GE Healthcare).

#### Antibodies and drugs.

Antibodies used for the immunoblotting and immunohistochemistry were phospho-p38 MAPK (Cell Signaling), p38 MAPKα, p38 MAPKγ, total p38 MAPK (Cell Signaling), phospho- and total ATF-2 (Cell Signaling), β-actin (Abcam), phospho-STAT3, total STAT3, and α-smooth muscle actin (Dako).

The p38 MAPK antagonist SB203580 was obtained from Selleck Chemicals and the dose used was 20 mg/kg given intraperitoneally once daily. The p38 MAPK antagonist PHA-00797804 was used with permission from Pfizer. This was administered intraperitoneally at 3 mg/kg once daily. The difference in kinase activity and specificity between SB203580 and PH-797804 is as follows: SB203580 IC_50_: 50 nM, *K*_i_: 21 nM vs. PH-797804 IC_50_: 26 nM, *K*_i_: 3.9 nM.

#### Quantification of pulmonary vascular remodeling.

Pulmonary vascular remodeling was quantified by determining the degree of muscularization present in smaller pulmonary arterial vessels with a diameter <80 μm. Standard immunohistochemical techniques were used as described below with staining for α-smooth muscle actin. In each lung section, pulmonary arteries were characterized as completely muscularized (with complete occlusion or slit-like appearance of lumen), partially muscularized (with incomplete rings of smooth muscle), or nonmuscularized (with no smooth muscle present). The number of vessels in each category was determined by counting the total number in five random fields per slide and using three slides per animal. The vessels were then categorized by degree of muscularization. The individual assessor was blinded to the slides.

#### Immunohistochemistry.

##### IN VIVO MODELS.

Immediately after experimentation, the left lung was removed and perfusion fixed with 10% neutral buffered formalin. Following fixation the paraffin embedded lungs were sectioned for analysis. These sections were immunohistochemically stained for p38 MAPKα or α-smooth muscle actin using standard techniques.

##### HUMAN TISSUE.

Lung tissue from two female patients with idiopathic pulmonary arterial hypertension (IPAH) was obtained at the time of lung transplantation centre with local ethics approval REC Reference 11/NE/0291. These patients were in New York Heart Association Functional Class 4 and on optimal background therapy including parenteral prostanoid therapy. Material was fixed in 10% phosphate-buffered formalin by airway perfusion before being prepared for sectioning. Samples were selected from specimens that showed excellent tissue preservation. Control adult lung was obtained from patients who were undergoing routine cardiothoracic intervention, with no echocardiographical evidence of pulmonary hypertension. Sections were immunohistochemically stained for p38 MAPKα and phospho-p38 MAPK. Control tissue was obtained from patients undergoing lung resection for solitary lung nodules and had no evidence of pulmonary hypertension on echocardiography.

#### Enzyme-linked immunosorbent assay/cytokine array.

An IL-6 enzyme-linked immunosorbent assay (ELISA; R&D Systems) was used to analyze lung homogenates prepared as previously described. The protocol as per manufacturer's instructions was followed. The rat proteome profiler (R&D Systems) was used to analyze 29 cytokines/chemokines as per manufacturers instructions. This is a qualitative analysis rather than quantitative.

#### SYBR green quantitative PCR.

Gene expression of the pulmonary artery fibroblasts was analyzed using SYBR green quantitative PCR. RNA was isolated from the cells using RNAEasy purification kits (Invitrogen), and the purity was quantified using Nanodrop reader. Then these were reverse transcribed and real-time quantitative PCR performed using SYBR Green PCR Mastermix and primers for rat IL-6 (Integrated DNA Technologies) on Applied Biosystems ABI7500 PCR machine. These experiments were done in triplicate. Relative gene expression was quantified by comparison of the “Ct” method relative to β-actin as a housekeeping gene.

#### Data analysis.

For normally distributed values, responses were reported as means ± SE and Students *t*-test was used. The *n* value refers to the number of animals involved per experimental procedure. For multiple comparisons of means across different experimental groups, ANOVA was performed with Bonferonni post hoc analysis. Values of *P* < 0.05 were accepted as statistically significant.

## RESULTS

### p38 MAPK and the α-Isoform Is Important in Both In Vitro And In Vivo Experimental Models of Pulmonary Vascular Remodeling

#### In vitro: hypoxia.

**Fig. 1.**
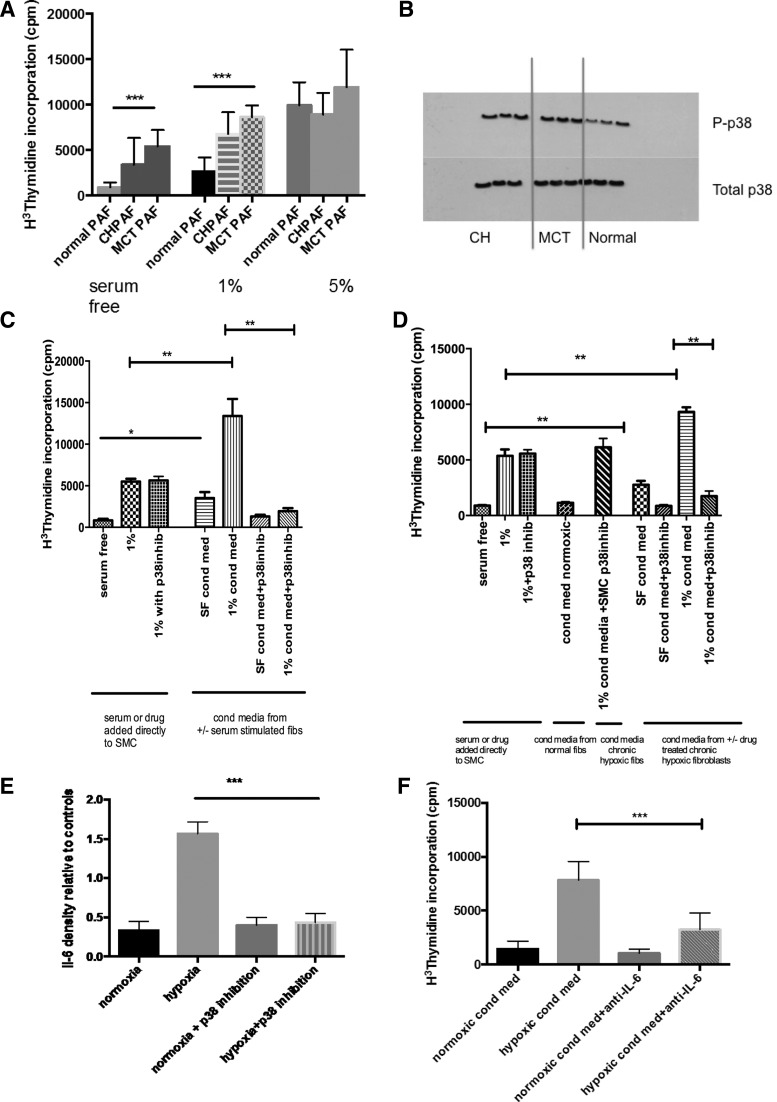
Proproliferative fibroblast phenotype and paracrine smooth muscle proliferation mediated by p38 MAPK in fibroblasts from experimental models of pulmonary hypertension (PH). *A*: cells were isolated from the 2nd order division of the pulmonary artery and grown in normoxic culture. The effect of serum stimulation was observed on these cells. At baseline with no serum stimulation, the chronic hypoxic (CH) and monocrotaline (MCT) pulmonary artery fibroblast (PAF) had an increased proliferative rate compared with the PAF derived from normoxic wild-type rats. This was also seen with low dose 1% serum stimulation. The effect was lost at 5% serum stimulation, which may reflect cell contact inhibition. The thymidine incorporation assay was used to assess the proliferation of the cells, and the results presented are representative of 3 experiments with triplicate values in each experiment. Values are means ± SE. ***P < 0.001. *B*: this immunoblot shows that under normoxic conditions the PAFs derived from both CH and MCT animals show a constitutive activation of p38 MAPK with increased levels of p-p38 detected. Cells were from passage 3 and were quiescesed in serum free medium for 24 h before harvest. Immunoblot for phospho-p38 and total p38 MAPK was performed and representative of 3 experiments. *C*: PAFs derived from MCT animals were isolated and then quiescesed for 24 h in serum free media. After 24 h, cells were stimulated with either 1% serum or some remained in serum free media ± SB203580 (p38 inhib). The conditioned media were aspirated from the wells after 24 h and passed through a cell sieve to give conditioned media from serum free cells ± SB203580 (SF cond med, SF cond med + p38 inhib) and 1% stimulated cells ± SB203580 (1% cond med, 1% cond med + p38 inhib). The conditioned media were added to smooth muscle cells for 48 h before a proliferation assay was performed. Both SF cond med and 1% cond med resulted in increased proliferation of pulmonary artery smooth muscle cells (PASMCs) while this effect was lost in the conditioned media from the PAFs, which had been coincubated with SB203580. There was no effect of SB203580 directly on the PASMCs. Values are means ± SE. Data shown from 3 experiments. **P < 0.005. *D*: PAFs derived from CH animals were isolated and then quiescesed for 24 h in serum free media. After 24 h, cells were stimulated with either 1% serum or some remained in serum free media ± SB203580 (p38 inhib). The conditioned media were aspirated from the wells after 24 h and passed through a cell sieve to give conditioned media from serum free cells ± SB203580 (SF cond med, SF cond med + p38 inhib) and 1% stimulated cells ± SB203580 (1% cond med, 1% cond med + p38 inhib). The conditioned media were added to normal PASMCs for 48 h before a proliferation assay was performed. Both SF cond med and 1% cond med resulted in increased proliferation of PASMCs while this effect was lost in the conditioned media from the PAF, which had been coincubated with SB203580. There was no effect of SB203580 directly on the PASMCs. Values are means ± SE. Data shown from 3 experiments. *P < 0.05; **P < 0.005. *E*: PAF from normal animals were exposed to 48 h normoxia or hypoxia ± SB203580. The supernatant was collected and analyzed using cytokine array. The above are representative of triplicate samples from 3 different animals. Exposure times for each blot was the same. The results for IL-6 are shown and are presented as a relative density plot vs. the positive control blots. ***P < 0.005. *F*: PASMCs are quiescesed and then incubated with conditioned media derived from normoxic or hypoxic PAF. The hypoxic conditioned media induced PASMC proliferation. When both the conditioned media and anti-IL-6 were added to the PASMCs, there was an inhibition in the proliferative stimulus. Results are means ± SE from 3 replicates from 3 different animals. ***P < 0.001.

Our group and others have shown previously that fibroblasts isolated from chronic hypoxic animals have undergone a phenotypic switch, which results in constitutive activation of p38 MAPK and a proproliferative phenotype. Whether this effect is seen in other models of pulmonary hypertension is unknown. Therefore, we examined the proliferative potential of fibroblasts derived from MCT animals and compared them to that of fibroblasts isolated from both normal and chronic hypoxic animals (Fig. 1*A*). This clearly showed an increased predisposition to proliferation by the MCT-derived fibroblasts in relation to low dose serum stimulation. The effect was lost at 5% serum stimulation, which may reflect cell contact inhibition.

We confirmed that there was increased phosphorylation of p38 MAPK in both chronic hypoxic and MCT fibroblasts compared with normal fibroblasts (Fig. 1*B*), suggesting upregulation of this pathway in those fibroblasts derived from experimental models of pulmonary hypertension. This was confirmed on densitometry (data not shown).

The addition of conditioned media isolated from chronic hypoxic and MCT fibroblasts to smooth muscle cells resulted in increased proliferation (Fig. 1, *C* and *D*). This effect could be prevented when the fibroblasts were incubated with a p38 MAPK inhibitor before collection of the conditioned media. In contrast p38 MAPK inhibition had no direct effect on pulmonary artery smooth muscle cell (PASMC) proliferation. This suggests that various mitogens are released from the fibroblast in a p38 MAPK-dependent mechanism, which can promote pulmonary vascular remodeling, and that this is conserved in fibroblasts derived from experimental models of pulmonary hypertension. To investigate this further a cytokine screening array was used (data not shown). Various cytokines were identified as being released into the supernatant from hypoxic fibroblasts and perhaps one of the most important with relevance to PVremod is IL-6. Other cytokines/chemokines identified were VEGF-A, CXCL-1, CXCL3, TIMP-1, LIX, and MIP-3.

With the addition of p38 MAPK inhibitor SB203580, the proinflammatory profile was returned to that of normoxic fibroblasts, suggesting a key role of p38 MAPK in release of proinflammatory mediators. The importance of IL-6 in the conditioned media were demonstrated by abrogation of the proliferative effect when the conditioned media were added in the presence of anti-IL-6 antibody (Fig. 1*F*). Although we identified other chemokines and cytokines, the significance of IL-6 in pulmonary vascular biology has already been shown and therefore seemed an appropriate signal to focus on.

#### In vivo: chronic hypoxia and MCT models.

**Fig. 2.**
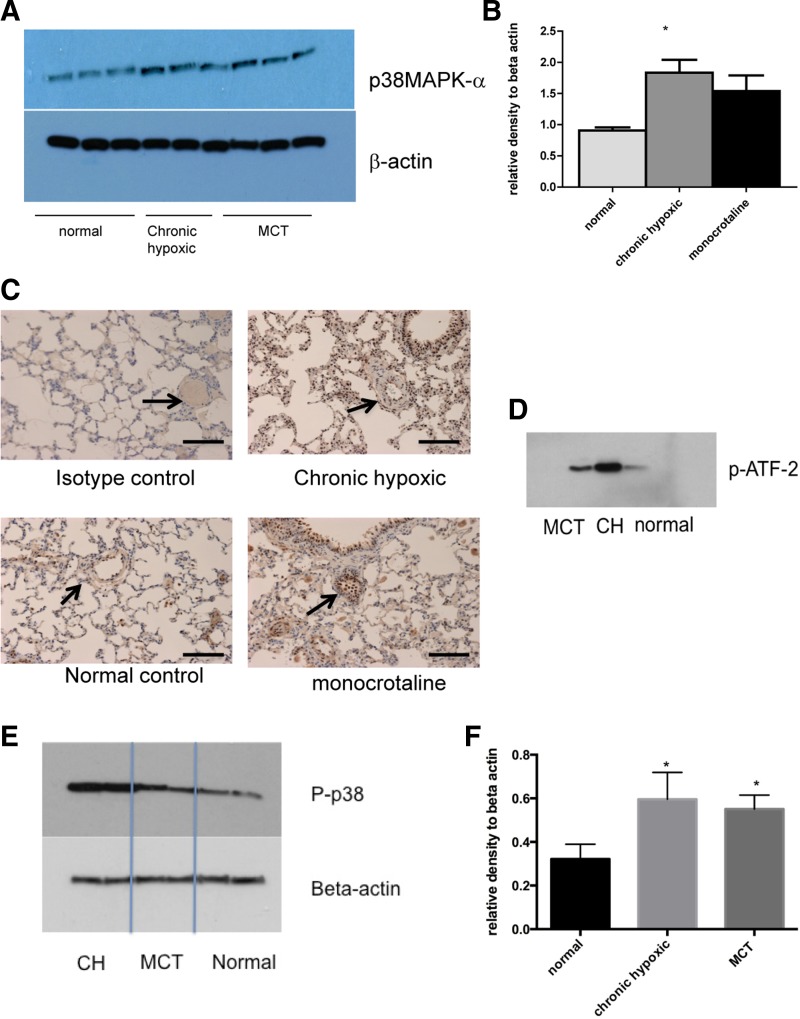
Increased expression of p38 MAPK α-isoform in animal models of PH. *A*: lungs from normal, CH, and MCT animals were harvested and homogenized with a cocktail of phosphatase and kinase inhibitors. The protein concentration was quantified using the BCA method. Equal concentrations were then loaded on a gel and blotted for p38 MAPKα and β-actin for loading control. There are 3 wells for each condition. Immunoblot shown is best representative of 3 experiments using 3 different animals with each condition. *B*: densitometry of immunoblot in *A*. Values are mean arbitrary values from 3 immunoblots expressed relative to the value for β-actin. *P < 0.05 by ANOVA. *C*: lung sections (5 mm) were prepared from normal, CH, and MCT animals. Sections were stained for p38 MAPKα using 1:400 dilution. Magnification: ×20. Bar represents = 150 mm. Arrows identify blood vessels. *D*: lungs from normal, CH, and MCT animals were harvested and homogenized with a cocktail of phosphatase and kinase inhibitors. The protein concentration was quantified using BCA method. Equal concentrations were then used in a p38 MAPK activity assay using immunoprecipitation and the phosphorylation of activating transcription factor-2 (ATF-2) as a read out. Immunoblot shown is representative of 3 experiments using 3 different animals. *E* and *F*: lungs from normal, CH, and MCT animals were harvested and homogenized with a cocktail of phosphatase and kinase inhibitors. The protein concentration was quantified using BCA method. Equal concentrations were then loaded on a gel and blotted for phosphorylated p38 MAPK and β-actin for loading control. Immunoblot shown is representative of 3 experiments using 3 different animals with each condition. Densitometry is shown for remaining blots. *P < 0.05.

Expression of p38 MAPKα was increased in both chronic hypoxic and MCT rat models of pulmonary hypertension after 2 wk (Fig. 2, *A* and *B*).

Immunohistochemistry showed increased p38 MAPKα in the small pulmonary vessels of both chronic hypoxic and MCT animals. This staining was distributed throughout the vessel wall with notable staining in the adventitial and endothelial compartments (Fig. 2*C*). With the use of a p38 MAPK activity assay, increased activity was demonstrated in whole lung homogenates from chronic hypoxic and MCT animals (Fig. 2*D*) suggesting because of increased expression there was also increased activity of the p38 MAPKα pathway. Furthermore, increased phosphorylated total p38 MAPK was observed in the lungs of those animals with pulmonary hypertension (Fig. 2, *E* and *F*). We did not find changes in expression of β- or γp38 MAPK isoforms (data not shown).

### Administration of the p38 MAPK Inhibitor SB203580 the Development of Chronic Hypoxic-Induced Pulmonary Hypertension

**Fig. 3.**
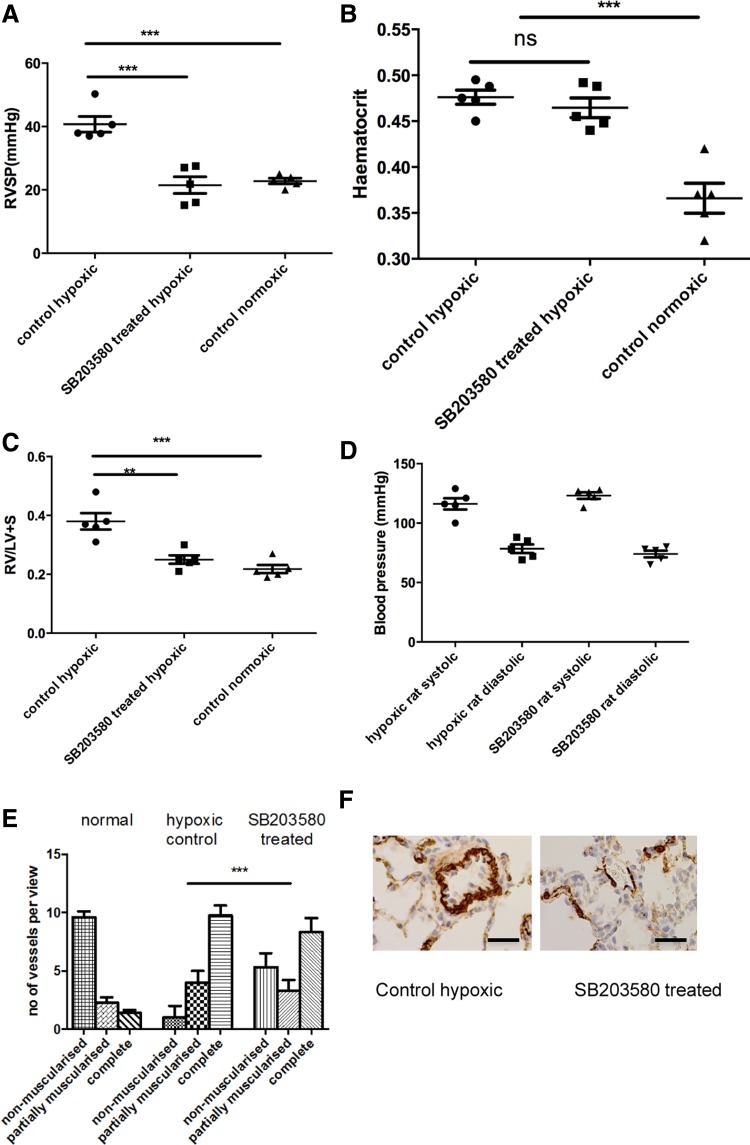
PH is prevented by administration of SB203580, a p38 MAPKα inhibitor. *A* and *B*: animals were exposed to a hypobaric hypoxic environment for 2 wk. Some animals received daily injections of SB203580. Hemodynamics (*A*) and hematocrit (*B*) were measured after 2 wk. RVSP, right ventricular (RV) systolic pressure. Data represent mean values ± SE. Total animals *n* = 5–6 per group. *C*: hearts were isolated from the animals and the RV was dissected out from the left ventricle (LV) and septum. The ventricles were dry blotted and then weighed, and the ratio was calculated. The total RV weight was also plotted. Values are means ± SD; *n* = 5 per group. **P < 0.05. *D*: noninvasive systemic blood pressure taken by tail-cuff measurement. *E* and *F*: rats were exposed to hypoxia ± SB203580. The lungs were removed after experiment and sections (5 mm) cut. These were stained with α-smooth muscle actin and the vessels <80 mm were analyzed for degree of muscularization. Five random fields per slide were analyzed with 3 slides per animal. The vessels were categorized as completely, partially, or nonmuscularized and are presented as numbers per total number of vessels present in each field. Groups analyzed by ANOVA for overall change with posttest analysis; *n* = 5 animals. ***P < 0.001, for *A*–*C* and *E*.

We administered daily p38 MAPKα-specific pharmacological inhibitor (SB203580) or vehicle (DMSO) via intraperitoneal injections to animals maintained in a hypoxic environment. After 14 days RV systolic pressure (RVSP) and RVH were significantly increased (*P* < 0.005) in the vehicle-treated animals but remained normal in the animals with the p38 MAPK inhibitor (Fig. 3, *A*–*D*).

There was a significant increase in the percentage of fully muscularized vessels in the vehicle group compared with the normal animals. This increase was significantly less in the drug-treated group indicating prevention of PVremod (Fig. 3, *E* and *F*). Knockdown of p38 MAPK activity in the lungs was confirmed by looking at ATF-2 phosphorylation (data not shown).

### The p38 MAPK Inhibitor SB203580 Reversed Chronic Hypoxic and MCT-Induced Pulmonary Hypertension

#### Chronic hypoxic pulmonary hypertension.

**Fig. 4.**
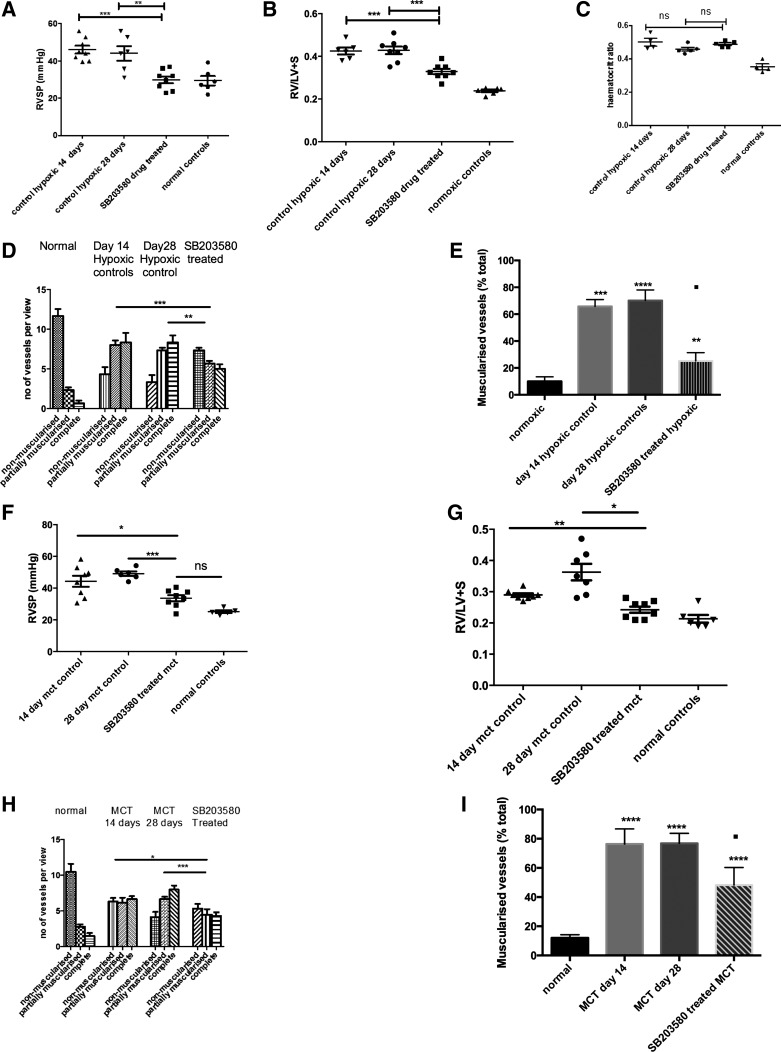
PH in 2 in vivo animal models is reversed by the administration of SB203580, a p38 MAPKα inhibitor. *A*: animals were exposed to a hypobaric hypoxic environment for 2 wk and then p38 MAPK inhibition was commenced. Hemodynamics and RVSP were measured after 4 wk. Data represent mean values ± SE. Total animals *n* = 5–6 per group. **P < 0.01; ***P < 0.001, for normal relative to all other conditions. *B*: hearts were isolated form the animals and the RV dissected out from the LV and septum. The ventricles were dry blotted and then weighed, and the ratio was calculated. Values are means ± SE; *n* = 6–7. **P < 0.01; ***P < 0.005. *C*: hematocrit ratio. *D*: the lungs were removed after experiment and sections (5 mm) cut. These were stained for α-smooth muscle actin and the vessels <80 mm were analyzed for degree of muscularization. Five to 10 random fields were analyzed with 3 slides per animal. The vessels were categorized as completely, partially, or nonmuscularized. Groups analyzed by ANOVA for overall change with posttest analysis; *n* = 6 animals. **P < 0.01; ***P < 0.001. *E*: the lungs were removed after experiment and sections (5 mm) cut. These were stained for α-smooth muscle actin and the vessels <100 mm were analyzed for degree of muscularization. Five to 10 random fields (×40) were analyzed with 3 slides per animal. The vessels were categorized as muscularized or nonmuscularized and the percentage of muscularized vessels calculated. Groups were analyzed by ANOVA for overall change with posttest analysis; *n* = 7 animals. ****P < 0.0001; ■*P* < 0.05 for hypoxic drug-treated vs. *day 14* hypoxic control. **P < 0.01; ***P < 0.001. *F* and *G*: animals were injected with MCT and after 2 wk p38 MAPK inhibition was commenced with daily injections. Hemodynamics and RVH were measured after 4 wk. Data represent mean values ± SE. Total animals *n* = 6–7 per group. *P < 0.05; ***P < 0.001, for *F*. *P < 0.05; **P < 0.01, for *G*. *H*: the lungs were removed after experiment and sections (5 mm) cut. These were stained for α-smooth muscle actin, and the vessels <80 mm were analyzed for degree of muscularization. Five to 10 random fields (×40) were analyzed with 3 slides per animal. The vessels were categorized as completely, partially or nonmuscularized. Groups were analyzed by ANOVA for overall change with posttest analysis; *n* = 6 animals. *P < 0.05; ***P < 0.001. *I*: lungs were removed after experiment and sections (5 mm) cut. These were stained for α-smooth muscle actin, and the vessels <100 mm were analyzed for degree of muscularization. Five to 10 random fields (×40) were analyzed with 3 slides per animal. The vessels were categorized as muscularized or nonmuscularized, and the percentage of muscularized vessels was calculated. Groups were analyzed by ANOVA for overall change with posttest analysis; *n* = 6 animals. ****P < 0.0001; ■*P* < 0.05 for hypoxic drug-treated vs. *day 14* MCT control.

Animals were exposed to hypoxia for a period of 2 wk and then treated with daily intraperitoneal injections of SB203580 or vehicle for a further 2 wk while remaining in hypoxia. There was a significant reduction in the RVSP in the drug-treated group compared with the vehicle-treated group at 4 wk and control hypoxic animals after 2 wk (Fig. 4*A*), with almost full reversal to that of normal animals.

The degree of RVH was reversed significantly (by 29%) in the drug-treated animals (Fig. 4*B*). The degree of PVremod was also reduced in the drug-treated animals compared with the control untreated animals (Fig. 4, *C* and *D*). The systemic blood pressure (BP) was unchanged between groups although the BP was lower in the 28-day hypoxic group (data not shown).

#### MCT-induced pulmonary hypertension.

Two weeks after an injection of MCT, there was an elevated RVSP and increased RVH consistent with the development of pulmonary hypertension in this group of animals. (Fig. 4, *E* and *F*). There was no difference between the RVSP rise seen in MCT control animals at 14 or 28 days.

After 14 days, these animals were injected daily with vehicle or SB203580 for a further 2 wk. This led to a significant reduction in the RVSP compared with both the 14- and the 28-day time points in MCT controls (Fig. 4*E*). There was a significant improvement in the degree of RVH observed in the drug-treated animals which almost returned to normal (Fig. 4*F*). This was mirrored by a reduction in the degree of muscularization in the small vessels in the drug-treated animals (Fig. 4, *G* and *H*). There was no difference in the systemic BP between the MCT groups (data not shown).

### Reversal of Chronic Hypoxic and MCT-Induced Pulmonary Hypertension with a Novel p38 MAPKα-Selective Inhibitor (PH-797804)

To examine the hypothesis that the α-isoform is important in the pulmonary hypertensive phenotype, we utilized a novel agent with greater specificity for this isoform than SB203580 (3).

#### Chronic hypoxic animals.

**Fig. 5.**
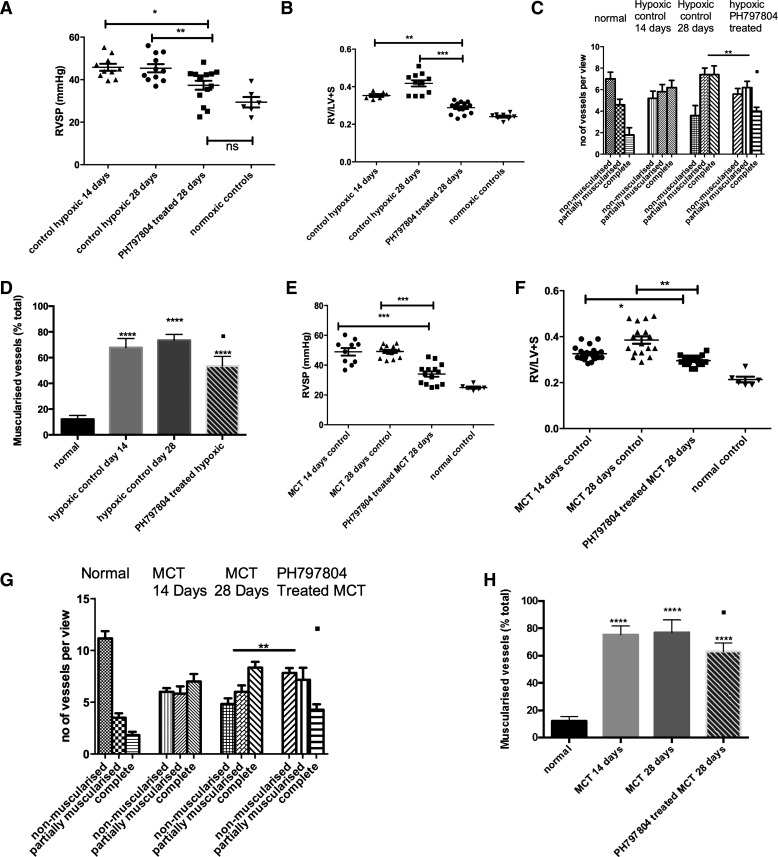
PH in a reversal strategy in 2 in vivo animal models by administration of PH-797804, a more selective p38 MAPKα inhibitor. *A* and *B*: animals were exposed to CH and after 2 wk p38 MAPK inhibition was commenced with daily injections. Hemodynamics and RV hypertrophy (RVH) were measured after 4 wk. Data represent mean values ± SE. *P < 0.05; **P < 0.01 for *A*. **P < 0.01; ***P < 0.001, for *B*. *C*: the lungs were removed after experiment and sections (5 mm) were cut. These were stained with α-smooth muscle actin and the vessels <80 mm were analyzed for degree of muscularization. Five to 10 random fields (×40) were analyzed with 3 slides per animal. The vessels were categorized as completely, partially, or nonmuscularized. Groups analyzed by ANOVA for overall change with posttest analysis; *n* = 10 animals. **P < 0.01; ■*P* < 0.05 for complete muscularized group in drug-treated vs. *day 14* hypoxic control. *D*: lungs were removed after experiment and sections (5 mm) cut. These were stained with α-smooth muscle actin and the vessels <100 mm were analyzed for degree of muscularization. Five to 10 random fields (×40) were analyzed with 3 slides per animal. The vessels were categorized as muscularized or nonmuscularized and the percentage of muscularized vessels calculated. Groups analyzed by ANOVA for overall change with posttest analysis; *n* = 10 animals. ****P < 0.0001; ■*P* < 0.05 for hypoxic drug-treated vs. *day 14* hypoxic control. *E* and *F*: animals were injected with MCT and after 2 wk p38 MAPK inhibition was commenced with daily injections. Hemodynamics and RVH were measured after 4 wk. Data represent mean values ± SE. Total animals *n* = 14–15 per group. ***P < 0.001 for *E*. *P < 0.05; **P < 0.01 for *F*. *G*: the lungs were removed after experiment and sections (5 mm) cut. These were stained with α-smooth muscle actin and the vessels <80 mm were analyzed for degree of muscularization. Five to 10 random fields (×40) were analyzed with 3 slides per animal. The vessels were categorized as completely, partially or nonmuscularized. Groups analyzed by ANOVA for overall change with posttest analysis; *n* = 10 animals. **P < 0.01; ■*P* < 0.05, for complete muscularized group in drug-treated vs. day 14 hypoxic control. *H*: lungs were removed after experiment and sections (5 mm) cut. These were stained with α-smooth muscle actin, and the vessels <100 mm were analyzed for degree of muscularization. Five to 10 random fields (×40) were analyzed with 3 slides per animal. The vessels were categorized as muscularized or nonmuscularized, and the percentage of muscularized vessels was calculated. Groups analyzed by ANOVA for overall change with posttest analysis; *n* = 10 animals. ****P < 0.0001; ■*P* < 0.05 for drug-treated vs. *day 14* MCT control.

We adopted a reversal strategy whereby the animals were treated daily intraperitoneal injections of PH-797804 after 2 wk of chronic hypoxia. Similar to the study with SB203580 we found a significant reduction in the RVSP in the drug-treated animals after 2 wk of treatment (Fig. 5*A*) accompanied by a marked reduction in the degree of RVH compared with both 14- and 28-day time points (Fig. 5*B*), with no change in hematocrit (data not shown). The pulmonary vascular muscularization was reversed in the drug-treated group compared with the 28-day animal untreated group (Fig. 5, *C* and *D*). Systemic BP remained unchanged. (data not shown).

#### MCT animals.

PH-797804 was shown to improve the RVSP in animals with established MCT-induced pulmonary hypertension (Fig. 5*E*). There was an improvement in the RVH in the drug-treated animals compared with the 14- and 28-day time points (Fig. 5*F*). Attenuation of progression in the degree of muscularization of the vessels in the drug-treated animals compared with the *day 28* untreated animals was identified, and there was a reduction of muscularization in the treated group compared with the *day 14* untreated animals, as shown Fig. 5*G*. Furthermore when the percentage of muscularized vessels was calculated and expressed as percentage of the total number of vessels, this demonstrated that there was a significant reduction in the overall degree of muscularization compared with *day 14* MCT (Fig. 5*H*). Again the systemic BP was unaltered (data not shown).

### p38 MAPK Inhibition Reduced Hypoxia-Induced Release of IL-6 from Pulmonary Artery Fibroblasts

**Fig. 6.**
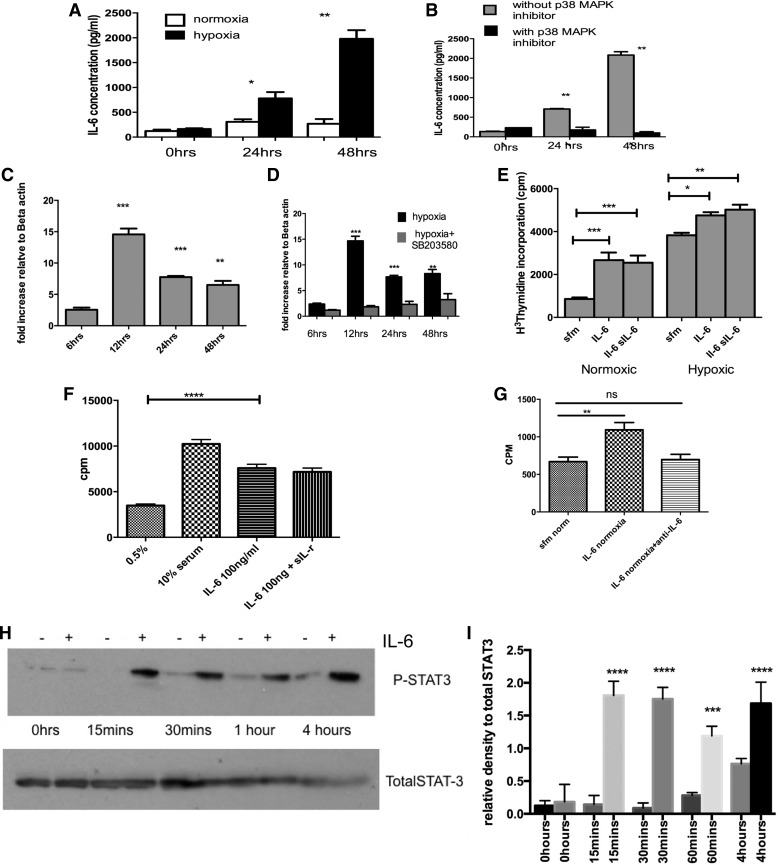
p38 MAPKα inhibition reduces interleukin (IL)-6 generation and signaling as a potential mechanism for beneficial effects in PH. *A* and *B*: supernatant from normoxic and hypoxic PAF ± SB203580 was analyzed using a quantitative ELISA to determine actual amounts released. Cells were growth arrested in serum free media for 24 h before hypoxic exposure. The values are mean ± SE values from triplicate wells for each sample and experiment repeated 3 times using cells from 3 animals. *P < 0.05 **P < 0.005. *C*: PAF were exposed to hypoxia and then RNA isolated after 48 h. The RNA was reverse transcribed into cDNA and then analyzed using quantitative (q)RT-PCR. The mRNA increased and peaked at 12 h of hypoxia. Values represent ratio of increase of IL-6 gene mRNA relative to the housekeeping gene of β-actin and calculated using comparative Ct method. Values are representative of triplicate samples from 3 experiments using 3 different animals. *D*: PAF were exposed to hypoxia in the presence of SB203580 and then RNA isolated after 48 h. The RNA was reverse transcribed into cDNA and then analyzed using qRT-PCR. The mRNA increased and peaked at 12 h of hypoxia. Values represent ratio of increase of IL-6 gene mRNA relative to the housekeeping gene of β-actin and calculated using comparative Ct method. Values are representative of triplicate samples from 3 experiments using 3 different animals. **P < 0.01; ***P < 0.01, for *C* and *D*. *E*: PAF were isolated and incubated with IL-6 (100 ng/ml) ± soluble IL-6 receptor (sIL-6) and then using DNA synthesis as a marker of cell proliferation, the response was observed. Data are mean values ± SE and are representative of duplicate experiments performed on cells from 3 different animals. *P < 0.05; **P < 0.005; ***P < 0.0001. *F*: PASMCs were exposed to IL-6 (100 ng/ml) ± soluble IL-6 receptor (sIL-r) and then using DNA synthesis as a marker of cell proliferation, the response was observed. Data are means ± and are representative of duplicate experiments performed on cells from 3 different animals. ****P < 0.0001. *G*: PASMCs were growth arrested for 24 h and then incubated with serum free media, IL-6 or IL-6 and anti-IL-6 antibody. Thymidine assay was used to quantify DNA synthesis, a measure of cell proliferation. Results are plotted as counts per million. Values are means ± SE and represent mean of 3 experiments on cells from same animal. A total of 3 different animals were used. **P < 0.01. *H* and *I*: PASMCs were stimulated with 100 ng/ml IL-6 (+) or without (−) and the protein harvested at baseline, 15 min, 30 min, 1 h, and 4 h. The cell lysates were immunoblotted for phosphorylated STAT3 and total STAT3. Experiment was repeated 3 times; blots above are representative of those experiments. *H* shows densitometry from repeat blots. ***P < 0.005; ****P < 0.001, for *I*.

Exposure of RPAF to hypoxia for up to 48 h led to upregulation of IL-6 and release of IL-6 from the cells. Secreted levels of IL-6 were significantly increased at 48 h of exposure. Treatment of cells with SB203580 prevented the release of the IL-6 from hypoxia-exposed fibroblasts (Fig. 6, *A* and *B*). This was time dependent with peak mRNA effect observed at 12 h after hypoxic exposure (Fig. 6*C*). This was reduced in the presence of p38 inhibition (data not shown). Further studies showed that pulmonary artery fibroblasts proliferate in response to IL-6, which was not significantly increased in the presence of the soluble IL-6R-α subunit (testing transactivation of the IL-6 pathway) but was more pronounced under hypoxic conditions (Fig. 6*D*). In PASMCs, the addition of IL-6 also proved to be a strong mitogen (Fig. 6*E*) and incubation with anti-IL-6 blocked the PASMC proliferation (Fig. 6*F*). In addition, the proliferation via IL-6 seemed to be mediated by the classical STAT-3 pathway with increased phosphorylation in a time-dependent manner as shown (Fig. 6, *G* and *H*).

### In vivo p38 MAPKα Inhibition Reduces Production of Tissue and Circulating IL-6 and Reverses Proproliferative Phenotype of Fibroblast

**Fig. 7.**
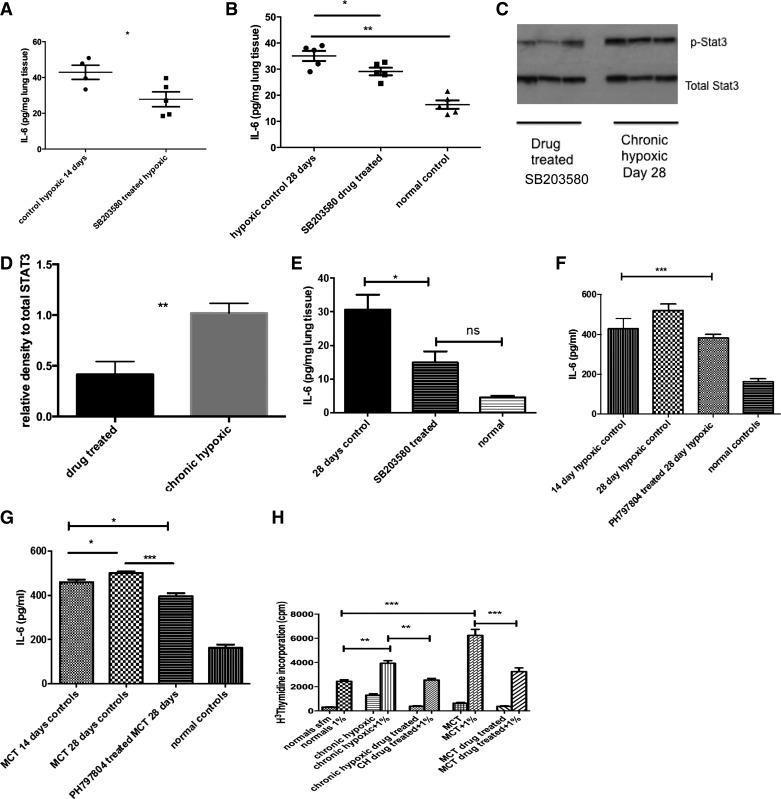
p38 MAPK inhibition in vivo leads to reduced IL-6 in experimental models of PH. *A*: lungs were isolated from CH animals in the prevention study with SB203580 and homogenized. The protein concentration was normalized by protein concentration as per BCA method. ELISA was used to analyze for IL-6 levels in the lung tissue. Data shown are means ± SE from triplicate samples from 4/5 animals in each group. *B*: lungs were isolated from normal and CH animals after treatment in reversal strategy with SB203580 and homogenized. The protein concentration was normalized by protein concentration as per BCA method. ELISA was used to analyze for IL-6 levels in the lung tissue. Drug-treated and normal control animals are at 28 days. Data shown are means ± SE from triplicate samples from 4/5 animals in each group. *P < 0.05 **P < 0.01. *C* and *D*: lungs from CH 28-day controls and SB203580-treated hypoxic animals were harvested and homogenized with a cocktail of phosphatase and kinase inhibitors. The protein concentration was quantified using BCA method. Equal concentrations were then loaded on a gel and blotted for phospho-STAT3 and total STAT3. Immunoblot shown is best representative of 3 experiments using lungs from 3 different animals with each condition. Densitometry is shown of other blots. **P < 0.001. *E*: lungs were isolated from MCT and normal control animals and homogenized. Inhibitor used was SB203580. The protein concentration was normalized by protein concentration as per BCA method. ELISA was used to analyze for IL-6 levels in the lung tissue. Data shown are means ± SE from triplicate samples from 4/5 animals in each group. *P < 0.05; ns is not significant by ANOVA. *F*: PH-797804 reduces serum IL-6 in reversal of CH-induced PH. Serum was collected from animals at the time of cardiac puncture and stored at −80°C until analysis could be performed. ELISA for IL-6 was performed on serum samples. Values shown are means ± SE. Samples were analyzed in duplicate and total animal number *n* = 11. ***P < 0.005. *G*: PH-797804 reduces serum IL-6 in reversal of MCT-induced PH. Serum was collected from animals at the time of cardiac puncture and stored at −80°C until analysis could be performed. ELISA for IL-6 was performed on serum samples. Values shown are means ± SE. Samples were analyzed in duplicate and total animal number *n* = 12. *P < 0.01; ***P < 0.005. *H*: fibroblasts undergo phenotypic switch back to normal after p38 MAPK inhibition. PAF were cultured from pulmonary arteries derived from normal, experimental models of PH (CH and MCT) and from animals after treatment with p38 MAPK inhibition PH-787904 for 2 wk. Cells were challenged with or without serum to assess proliferation; *n* = 3–4 per group. Experiment repeated 3 times. **P < 0.01, ***P < 0.001.

In the prevention study of the chronic hypoxic-associated pulmonary hypertension we found a reduction in IL-6 levels in the lungs of SB203580 drug-treated animals compared with vehicle-treated hypoxic controls (Fig. 7*A*). Similarly, in the reversal study with SB203580, IL-6 levels were reduced in the lung and the amount of phospho-STAT3 signaling was reduced (Fig. 7, *B*–*D*).

The same findings were observed in the MCT animals with reduced IL-6 levels in the SB203580 drug-treated animals (Fig. 7*E*). In the reversal studies using PH-797804, serum levels were examined and a significant reduction in serum IL-6 level was seen in the chronic hypoxic and MCT reversal study (Fig. 7, *F* and *G*). Other cells such as neutrophils can act as IL-6 reservoirs, and it is likely that the fibroblast is not the only source of IL-6. However, initial experiments have shown no increase in neutrophil activity in our models (data not shown).

Of interest is that the fibroblasts isolated from the pulmonary artery of the experimental models following treatment with p38 inhibition, no longer demonstrated the proproliferative phenotype previously observed (Fig. 7*H*). This suggests that in the experimental pulmonary hypertension fibroblast blockage of the p38 MAPK pathway can restore normal phenotype to the cell in vivo. This may be one of the factors leading to the reversal of PVremod in the drug-treated animals.

### Increased Expression of phospho-p38 MAPK and p38 MAPKα in Vessels from Human IPAH Patients

**Fig. 8.**
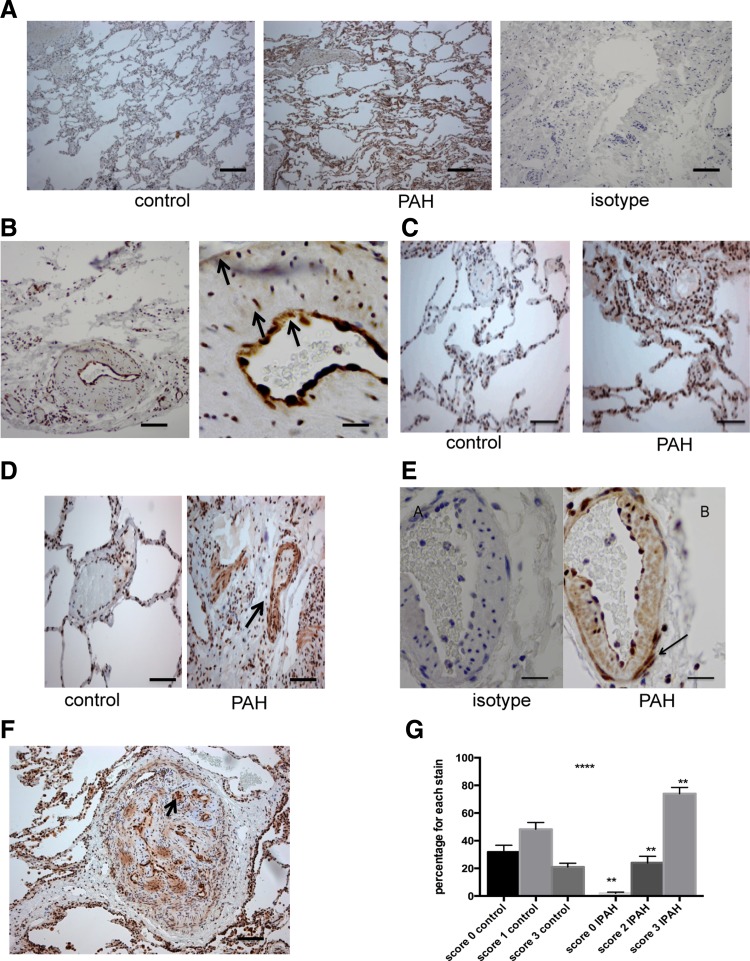
Phospho-p38 MAPK and p38 MAPKα expression in explanted lungs from patients with idiopathic pulmonary arterial hypertension (IPAH). *A*: sections of 5 mm were taken. Then, normal control lung and IPAH lung are stained for phospho-p38 MAPK at dilution of 1:300. The isotype on IPAH lung is also shown. This dilution was optimally assessed for. Objective: ×20. Bar = 150 mm. *B*: high-power microscopy shows that there is strong staining for phospho-p38 MAPK in the intima, media, and the adventitia (arrows). Objective lens: ×20 and ×40. Bar = 50 mm. *C*: sections of control lung (*A*) and IPAH lung (*B*) were stained for p38 MAPKα at dilution of 1:300. Objective lens × 20. Bar = 150 mm. *D*: staining for p38 MAPKa showed increased cytosolic staining in the IPAH lung (*right*) compared with control lung (*left*). *E*: high-power view (×40) of staining with isotype and p38 MAPKα in a vessel in IPAH lung. This shows staining throughout the vessel layers but especially in adventitia and fibroblast cells (arrow). Bar = 50 mm. *F*: low-power view of a plexiform lesion. Staining for p38 MAPKα using 1:300 dilution. Objective lens × 20. Bar = 150 mm. *G*: histological scoring shows increased p38 MAPKα staining throughout the vascular wall. With the use of a well-validated histological scoring system (Allred), the vascular wall cells were scored for intensity of staining. The intensity multiplied by the number of vessels with that intensity determines the values. Values shown are from 5 random high-power fields from 2 slides. ****P < 0.0001 by ANOVA. **P < 0.001 for individual IPAH vs. control columns.

Immunohistochemical staining of sections from the lungs of patients with IPAH who had undergone transplantation showed increased phospho-p38 MAPK expression compared with control lungs (Fig. 8*A*). This is shown in all layers of the vascular wall (Fig. 8*B*). There was increased staining for p38 MAPKα, which was mainly in a nuclear distribution in the control vessels but involved both nuclear and cytoplasmic staining in the IPAH patients (Fig. 8, *C* and *D*). This was again demonstrated in all the layers of the vascular wall (Fig. 8*E*). With the use of an intensity scoring technique, there was higher expression demonstrated for p38 MAPKα in the vessels of patients with IPAH compared with controls (Fig. 8*F*) (1).

In the identified plexiform lesion, there was strong cellular staining for p38 MAPKα in cells around the vascular channels in both cytosol and nucleus (Fig. 8*G*).

## DISCUSSION

This study advances our understanding of the potential role that p38 MAPK plays in the biology of pulmonary vascular remodeling. It demonstrates that p38 MAPKα is implicated in the pathogenesis of pulmonary hypertension and administration of selective p38 MAPK inhibitors in vivo demonstrated that the pulmonary hypertensive phenotype cannot only be prevented, but reversed, with improvements in PVremod, acting through inhibition of key inflammatory mediators such as IL-6. Although p38 MAPK has been implicated previously in cytokine biosynthesis and to have a role in MCT-induced pulmonary hypertension, this study is the first to show that the α-isoform is specifically implicated and inhibition can lower IL-6 levels and reverse disease. This suggests there may be a novel link between p38 MAPK signaling and inflammation in the development of pulmonary vascular remodeling.

The fibroblast is an important cell in the pathobiology of pulmonary vascular remodeling and dysregulation of the p38 MAPK pathway in the fibroblast can result in aberrant proliferation. We have previously shown, along with others, that the fibroblast undergoes a proproliferative phenotypic switch when exposed to chronic hypoxia and that p38 MAPK is critically involved. Here we have shown a similar proproliferative transformation with the fibroblasts isolated from MCT animals, again mediated through p38 MAPK. Conditioned media isolated from the hypoxic fibroblast can stimulate smooth muscle cell proliferation and using cytokine arrays, IL-6 was identified as a potential key mediator that was being released by the fibroblast under hypoxic conditions. IL-6 release was blocked by the p38 MAPK inhibitor SB203580 in fibroblasts, implicating p38 MAPK in both proliferation and cytokine biosynthesis in this cell. Similar findings were obtained from conditioned media isolated from fibroblasts derived from experimental animal models of pulmonary hypertension. Therefore, our in vitro fibroblast studies showed that under acute hypoxic conditions there was increased IL-6 production and that IL-6 can, through a STAT-3 pathway, stimulate proliferation of the smooth muscle cell and fibroblast.

Increased activity of p38 MAPK in both chronic hypoxic and MCT lung homogenates is in concordance with a previous study showing increased activity of the entire MAPK signaling group in large pulmonary arteries of chronically hypoxic rats (5). However, that study did not specifically demonstrate whether p38 MAPK activity itself was increased or address α-isoform expression. Here, we have shown evidence that p38 MAPK and the α-isoform are upregulated in the pulmonary vessels of two different in vivo animal models of pulmonary hypertension and has been implicated as a proproliferative and proinflammatory pathway in the pulmonary artery fibroblast.

Using the p38 MAPK inhibitor SB203580 we showed that blocking p38 MAPK could prevent the development of pulmonary hypertension as previously described in a MCT study that looked at prevention of the disease only (8). Although prevention studies are critical in outlining early pathogenic mechanisms, studies with established disease are more clinically relevant. In this study after development of established pulmonary hypertension in both hypoxic and MCT models, daily administration of SB203580 reversed the RVSP and RVH and also improved PVremod.

The novel and specific p38 MAPKα inhibitor PH-797804 has been shown to be extremely effective in animal models of rheumatoid arthritis with a 6.9-fold increase in specificity relative to the p38 MAPK β-isoform compared with existing inhibitors (3). Furthermore, it has shown tolerability, safety, and efficacy in a clinical trial of patients with chronic obstructive pulmonary disease (9). This allowed us to use a drug that has the potential for future development in the treatment of patients with pulmonary hypertension. In addition the targeting of the more specific isoform differentiates the systemic and pulmonary circulation. The p38 MAPK pathway seems to be preferentially upregulated in the pulmonary circulation in pulmonary hypertension and hence provides a rationale for targeting of this pathway. There is support from a study looking at bleomycin-induced pulmonary fibrosis in rats. This showed that inhibition of p38 MAPK can prevent the development of RVH, although this was a prevention study in an animal model not representative of true pulmonary arterial disease (18). Although no animal model fully recapitulates disease, the models employed in this study are established and recognized models of pulmonary arterial hypertension.

Our data with PH-797804, performed in reversal in vivo studies, produced similar findings to SB203580 with a reduction in the RVSP and RVH and significant attenuation of PVremod in the chronic hypoxic model. There was improvement in the RVSP in the drug-treated MCT group and a significant reversal and attenuation in progression of RVH. The reason that there was a greater observed reduction in the remodeling in the SB203580-treated animals may reflect that the dose used for PH-797804 was not optimal for this compound or that the length of administration was not long enough. The dose and length of administration for SB203580 in other animal models have been clearly identified. However, one of the novel aspects of this study is that using a clinically available, specific p38 MAPKα inhibitor leads to reversal of pulmonary vascular remodeling in two well-established animal models of pulmonary hypertension.

Cytokines and inflammatory responses have been recognized as a driver for PVremod and p38 MAPK has been implicated in inflammatory processes. IL-6 is a known mitogen for vascular smooth muscle cells, and previous transgenic murine studies have implicated IL-6 in the etiology of pulmonary hypertension (2, 10, 16). Moreover, a recent study has identified the upregulation and increased release of IL-6 from pulmonary artery fibroblasts derived from chronic hypoxic calves (7). In addition, serum levels of IL-6 correlate with the prognostic outcome in patients with IPAH (15).

Our in vitro studies showed the fibroblast to be a source of IL-6, and in both prevention and reversal studies we found a reduction in the concentration of IL-6 in the lungs and serum of drug-treated animals. Although likely not to be the only source of IL-6, the data presented here suggest the fibroblast can be an important source of this proinflammatory cytokine in the pulmonary vasculature. Studies on human umbilical vascular endothelial cells and alveolar macrophages have also shown that p38 MAPK inhibition can reduce IL-6 release (6, 18). However, our study has shown that inhibition of p38 MAPK resulted in a reversal of the proproliferative phenotype in the fibroblast isolated from in vivo-treated animals, suggesting that in experimental models inhibition can alter the underlying pathological process in the pulmonary vasculature. This study has shown modest reductions in lung IL-6 and serum levels of IL-6. Although not conclusive in these studies, it does raise the interesting possibility that p38 MAPK inhibition reduces local and serum IL-6 levels and that this may underplay a key role in the reversal of pathological remodeling.

Of major interest was our finding of increased activity of phosphorylated p38 MAPK and expression of p38 MAPKα in the vessels of explanted lungs from patients with IPAH. To our knowledge this is the first time this has been shown in pulmonary arterial hypertension. This was seen in both large and small vessels with increased nuclear and cytosolic distribution, involving endothelial, smooth muscle, and fibroblast cells, including the plexiform lesion, which is pathognomonic of pulmonary vasculopathy. The findings of p38 activation in all layers of the vasculature would suggest that it is unlikely that inhibition of p38 MAPK in the fibroblast alone is producing reversal of the experimental pulmonary hypertension. Indeed our previous work demonstrated that the endothelium in hypoxic pulmonary hypertension is associated with impaired relaxation and reduced nitric oxide production, which could be reversed by inhibition of p38 MAPK (19). However, our data clearly suggest that p38 MAPK is an important pathway throughout the pulmonary vasculature but that the activation of this pathway is critical in the fibroblast, a key cell in the process of PVremod.

In summary, the findings presented here suggest a critical role for p38 MAPK and the α-isoform in the development and progression of pulmonary hypertension. The fact that increased p38 MAPKα activity and expression are observed in both hypoxia and MCT animal models and in human disease suggests a common role in the etiology of many forms of pulmonary hypertension. Furthermore, p38 MAPK signaling through IL-6 provides a novel link between vascular remodeling and inflammation. This provides us with the exciting prospect of a new anti-inflammatory target for the treatment of pulmonary hypertension which improves the underlying remodeling process.

## GRANTS

This work was supported by a Wellcome Trust Clinical Training Fellowship.

## DISCLOSURES

No conflicts of interest, financial or otherwise, are declared by the author(s).

## AUTHOR CONTRIBUTIONS

A.C.C., R.M.W., G.B., A.J.F., D.J.W., and A.J.P. conception and design of research; A.C.C. and D.H.M. performed experiments; A.C.C., R.M.W., and D.J.W. analyzed data; A.C.C. and R.M.W. interpreted results of experiments; A.C.C. prepared figures; A.C.C., D.J.W., and A.J.P. drafted manuscript; A.C.C., R.M.W., D.J.W., and A.J.P. edited and revised manuscript; A.C.C., D.H.M., R.M.W., G.B., A.J.F., D.J.W., and A.J.P. approved final version of manuscript.
